# Barreras y estrategias para la accesibilidad a la salud de las personas trans en Córdoba, Argentina

**DOI:** 10.18294/sc.2025.5200

**Published:** 2025-02-05

**Authors:** María Laura Cordero, Lorena Saletti-Cuesta

**Affiliations:** 1 Magíster en Epidemiología, Gestión y Políticas de Salud. Programa de formación en Ley Micaela, Unidad Central de Políticas de Género, Universidad Nacional de Córdoba, Córdoba, Argentina. marialaura.cordero@gmail.com Universidad Nacional de Córdoba Programa de formación en Ley Micaela Unidad Central de Políticas de Género Universidad Nacional de Córdoba Córdoba Argentina marialaura.cordero@gmail.com; 2 Doctora en Salud, Antropología e Historia. Investigadora adjunta, Consejo Nacional de Investigaciones Científicas y Técnicas, con sede en el Centro de Investigaciones y Estudios sobre Cultura y Sociedad, Universidad Nacional de Córdoba, Córdoba, Argentina. lorenasaletti@unc.edu.ar Consejo Nacional de Investigaciones Científicas y Técnicas Consejo Nacional de Investigaciones Científicas y Técnicas Centro de Investigaciones y Estudios sobre Cultura y Sociedad Universidad Nacional de Córdoba Córdoba Argentina lorenasaletti@unc.edu.ar

**Keywords:** Servicios de Salud para las Personas Transgénero, Accesibilidad a los Servicios de Salud, Investigación Cualitativa, Diversidad, Equidad e Inclusión, Argentina, Health Services for Transgender Persons, Health Services Accessibility, Qualitative Research, Diversity, Equity and Inclusion, Argentina

## Abstract

Las trayectorias de vida de las personas transgénero están atravesadas por múltiples vulneraciones. Este trabajo pretende conocer la accesibilidad al sistema de salud de las personas trans en la provincia de Córdoba, Argentina. Desde un diseño narrativo y una aproximación al enfoque biográfico, entre 2022 y 2023 se realizaron ocho entrevistas semiestructuradas a personas trans para indagar sobre las barreras en el acceso a la salud y las estrategias que implementaron. De los relatos se desprende que, si bien la discriminación y los malos tratos continúan siendo la principal barrera, se cuestiona especialmente la falta de formación y de conocimientos sobre la salud trans por parte de profesionales. Como estrategias, se resalta la vinculación y el trabajo de los espacios colectivos y las organizaciones sociales LGBTTTIQ+ que son quienes facilitan el acceso a la salud. Las redes sociales para la transmisión de la información aparecen como estrategias claves en el acceso, al igual que los consultorios integrales inclusivos en los servicios de salud.

## Abstract (en)

The life trajectories of transgender individuals are marked by multiple vulnerabilities. This study aims to explore healthcare accessibility for trans people in the province of Córdoba, Argentina. Using a narrative design and a biographical approach, eight semi-structured interviews were conducted between 2022 and 2023 with trans individuals to examine the barriers to healthcare access and the strategies they employed. The accounts reveal that while discrimination and mistreatment remain the primary barriers, a notable concern is the lack of training and knowledge about trans health among healthcare professionals. Among the strategies highlighted are the involvement and efforts of collective spaces and LGBTTTIQ+ social organizations, which play a key role in facilitating access to healthcare. Social networks for disseminating information emerge as critical strategies, alongside inclusive comprehensive healthcare clinics within health services.

## INTRODUCCIÓN

Las múltiples situaciones de desigualdad, vulneraciones y violencias estructurales que viven las personas trans son reconocidas y difundidas a través de investigaciones científicas, entre otras voces, tanto a nivel mundial^1^^,^^2^^)^ como local. Por ejemplo, en Argentina, la violencia social, los obstáculos para el acceso a la educación, a la vivienda, al trabajo formal y a la salud constituyen desigualdades centrales, tal como refleja el *Primer Relevamiento Nacional de Condiciones de Vida de la Diversidad Sexual y Genérica en la Argentina*, que destaca la alta tendencia a la ideación suicida, depresión, ansiedad y estrés^3^.

Las personas autopercibidas como transgénero vivencian una identidad de género diferente a la asignada culturalmente al nacer según su sexo biológico. Esta identificación implica, en general, pero no siempre, la necesidad de atravesar transformaciones, muchas de ellas del propio cuerpo, modificando formas orgánicas, metabólicas y otros aspectos del cuerpo que varían según las necesidades individuales, los cuales requieren prácticas médicas, la mayoría de carácter invasivo. Así, cuando la persona trans así lo decide, se tornan imprescindibles las intervenciones de profesionales para que el proceso de construcción corporal no se convierta en un riesgo para la vida. En este sentido, el acceso a los sistemas de salud para las personas trans es de vital importancia y, como tal, constituye un derecho humano fundamental. 

La Ley 26743 de Identidad de Género, sancionada en Argentina en 2012, reconoce la autopercepción de la identidad y regula el acceso a la atención sanitaria integral, que incluye las intervenciones quirúrgicas y los tratamientos hormonales para adecuar el cuerpo a la identidad de género autopercibida^4^. Tal como señala Anahí Farji Neer, entre otros derechos, la ley posibilitó acceder a la hormonización y a las cirugías totales o parciales de adecuación corporal sin autorización judicial ni administrativa, cuando previamente se encontraban prohibidas por la Ley de Ejercicio de la Medicina y por el Código Penal^5^. El impacto de la ley en la atención sanitaria ha sido reconocido en la literatura científica^6^^,^^7^. La implementación de consultorios inclusivos y amigables, espacios de salud especializados para personas trans que reconocen la especificidad en los abordajes e intervenciones y la importancia de la perspectiva de género y de derechos humanos, es una estrategia reciente para facilitar el acceso a la salud^8^^,^^9^^,^^10^^,^^11^^,^^12^^,^^13^. Se encuentran en el primer nivel de atención o dentro de hospitales de segundo y tercer nivel de atención y están integrados por un equipo interdisciplinario y también por personal administrativo, agentes de salud, promotoras y promotores de salud que acompañan principalmente tratamientos de hormonización y controles de salud, y realizan articulaciones con otras áreas^14^. 

En la provincia de Córdoba, Argentina, el Programa Provincial de Acceso a la Salud Integral de personas LGBTTTIQ+, en 2024, contabilizó veintiocho centros: nueve en capital y diecinueve en otras localidades de la provincia^15^. Por otra parte, en 2021 se sancionó la Ley 27636 conocida como Ley de Acceso al Empleo Formal para personas Travestis, Transexuales y Transgéneros “Diana Sacayán-Lohana Berkins” que establece que al menos el 1% de los cargos estatales deben ser ocupados por personas travestis, transexuales y transgénero^16^, esta medida contribuyó a la inclusión formal en el empleo, y al acceso a obras sociales para la atención de la salud.

A pesar de estas políticas, el estigma, la discriminación y los malos tratos sistemáticos por parte del personal sanitario han generado que muchas personas trans solo acudan a los servicios de salud en situación de extrema gravedad o que implementen prácticas de construcción corporal invasivas sin acompañamiento profesional, con graves consecuencias para la propia salud^9^^,^^10^^,^^17^. 

En este trabajo comprendemos la salud como un derecho humano que debe ser garantizado por el Estado a través de políticas públicas que garanticen el efectivo goce del derecho a la salud a sus poblaciones, por ello es clave comprender la dinámica de los factores implicados en su acceso y disfrute^18^. En Argentina la esperanza de vida de las personas trans continúa siendo significativamente inferior al resto de la población, lo que devela que las desigualdades y vulnerabilidades, producto de las discriminaciones y violencias en las trayectorias de vida, aún no se han resuelto. Dentro de las principales causas de muerte de las personas transgénero se identifican las enfermedades asociadas a VIH y transfemicidios, y luego a cirrosis, sobredosis, problemas derivados de inyecciones de silicona y suicidio^19^. 

A nivel global, la literatura sobre la salud de las personas trans ha indagado sobre todo las barreras de acceso a la salud, especialmente, al proceso de construcción corporal^8^^,^^10^^,^^17^^,^^20^^,^^21^^,^^22^. En este sentido, se considera relevante poder estudiar, a través de las trayectorias de vida de las personas transgénero, el vínculo con las instituciones de salud, los conocimientos, tanto individuales como colectivos, y las redes que acompañan y sostienen la vida cotidiana de estas personas.

Entendemos la accesibilidad desde la concepción relacional y dinámica del concepto, aportada por Comes *et al*.^23^, quienes sostienen que las personas usuarias del sistema de salud también son generadoras de accesibilidad. Es decir, que tanto los servicios de salud y las personas tienen o no la posibilidad de encontrarse, en tanto están en una relación que no solo implica la accesibilidad geográfica, administrativa, económica, sino además la accesibilidad simbólica, el imaginario social y las representaciones tanto del personal de las instituciones como de las personas usuarias del sistema, aspectos claves tanto para el ingreso como para la permanencia en el sistema.

Desde esta concepción relacional de la accesibilidad, se analizan las barreras y las estrategias para el ingreso (accesibilidad inicial) y permanencia (accesibilidad ampliada) en el sistema de salud^23^. Las estrategias que las personas usuarias, en este caso personas transgénero, desarrollan para poder garantizarse salud, son primordiales para acceder al sistema. En este mismo sentido, entendemos por *estrategias* aquellas acciones, tanto individuales como colectivas, muchas de ellas históricas de las organizaciones y colectivos trans y otras más recientes vinculadas al uso de nuevas tecnologías, que de forma creativa y organizada posibilitan sortear y disminuir barreras existentes para acceder a la salud. Es por ello que el objetivo del presente trabajo es conocer la accesibilidad a los servicios de salud de las personas transgénero que residen en Córdoba indagando tanto las barreras como las estrategias de acceso.

## METODOLOGÍA

Se realizó un estudio cualitativo con un diseño narrativo, haciendo una aproximación al enfoque biográfico que busca construir narrativas a partir de relatos de experiencias de vida de las personas. Para ello considera la singularidad y la heterogeneidad de las situaciones individuales en búsqueda de conocer los elementos comunes y las coordenadas histórico-biográficas^24^. Desde este enfoque, se entiende que cada una de las trayectorias vitales reflejan una época, normas y valores sociales compartidos^25^. Este enfoque posibilita la reconstrucción de las historias de vida de las personas a partir del entrecruzamiento de tres dimensiones: 1) los elementos subjetivos (percepciones que tiene la persona sobre los diferentes aspectos de su propia vida) y objetivos (las instituciones sanitarias, educativas, políticas, la comunidad, el mercado de trabajo en el que se desarrolla la trayectoria de vida); 2) la influencia de los cambios en el contexto histórico y social, así como las variaciones a lo largo del ciclo vital de la persona; y 3) la articulación entre los elementos subjetivos y objetivos^25^. 

Cobran relevancia los movimientos y transiciones de las personas a lo largo de sus vidas, como así también los acontecimientos que, tanto por situaciones de la propia vida como externas vinculadas al período histórico, pueden operar como puntos de bifurcación en las trayectorias de vida, cambiando el rumbo^26^. Es decir, no solamente se consideran los acontecimientos de la vida personal, sino también hechos sociales, políticos, históricos que afectan la vida de las personas.

Se realizaron entrevistas biográficas semiestructuradas^27^ para conocer las trayectorias de vida con énfasis en indagar la salud, y orientando la entrevista para que el desarrollo de los aspectos indagados pueda ser relatado en forma cronológica, buscando conocer también los vínculos con redes y estrategias de acceso a la salud y al sistema de salud a lo largo de sus vidas. El guion de la entrevista se realizó sobre la base de la búsqueda de antecedentes sobre el tema. En las primeras entrevistas se corroboró la comprensibilidad y la pertinencia de las preguntas. 

La muestra fue por conveniencia, los criterios de inclusión fueron que la persona se identifique con una identidad trans, que resida en la provincia de Córdoba y que exprese voluntad para participar de la investigación. Se realizaron ocho entrevistas a personas trans en localidades cercanas a la ciudad de Córdoba y en la capital cordobesa. Las entrevistas fueron pautadas previamente vía telefónica con las personas participantes. Para contactarlas, se utilizó la técnica bola de nieve, partiendo de referentes claves que integran el equipo de salud del sector público de una localidad cercana a Córdoba y de una referente de una organización social, que trabaja por los derechos del colectivo LGBTTTIQ+, integrado en parte por personas transgénero. 

Las entrevistas se realizaron tanto en forma presencial, en el domicilio particular, en espacios públicos, en bares, como virtual mediante la utilización de plataformas como *meet* o videollamadas. Todas tuvieron una duración aproximada de una hora, fueron grabadas y posteriormente transcritas. Se realizó un análisis temático que permitió identificar y analizar temas dentro del corpus de datos obtenidos a partir de las entrevistas realizadas^28^. Una de las características principales es su flexibilidad^28^, necesaria para tomar decisiones al momento de analizar el material, teniendo en cuenta las particularidades que surgieron en cada entrevista. Se identificaron temas y puntos de bifurcación en la vida de las personas, desde el enfoque biográfico.

Con relación a los aspectos éticos de la investigación, en el primer contacto con las personas entrevistadas se compartió información de la propuesta de investigación y los criterios para la participación, asegurando la comprensión y la decisión libre de participar. Se compartió el consentimiento informado y se les informó sobre el resguardo de la confidencialidad, incluyendo el uso de pseudónimos y la eliminación de cualquier dato identificable en los resultados. Con relación a la participación de la persona de 16 años, según el Artículo 26 del Código Civil y Comercial de la Nación, en Argentina, las personas adolescentes a partir de los 16 años tienen capacidad plena para la toma de decisiones sobre el cuidado del propio cuerpo como persona adulta. Además, a partir de los 16 años, el o la adolescente puede peticionar todos los procedimientos que habilita la Ley de Identidad de Género, prescindiendo del requisito de mayoría de edad, de acuerdo al Artículo 26 del Código citado. Aún así, en la entrevista realizada participó como observadora no participante la madre de la persona entrevistada.

La confidencialidad fue abordada mediante el almacenamiento seguro de los datos en sistemas protegidos y accesibles solo al equipo de investigación, así como la anonimización de las entrevistas durante el análisis y la publicación de los resultados.

Este artículo se basa en la tesis de maestría titulada “Trayectorias de personas transgénero en el sistema de salud de las localidades de Unquillo y Córdoba, Argentina”, realizada en el marco de la Maestría en Epidemiología, Gestión y Políticas de Salud, del Instituto de Salud Colectiva, Universidad Nacional de Lanús, cuyo proyecto de investigación fue aprobado por el Comité de Ética en Investigación de la Universidad Nacional de Lanús.

## RESULTADOS

Se realizaron cuatro entrevistas a mujeres trans y cuatro a varones trans cuyas características sociodemográficas se recogen en la Tabla 1. 

**Tabla 1:** Caracterización sociodemográfica de la muestra, provincia de Córdoba, Argentina. 2022-2023.

Pseudónimos	Edad	Identidad de género	Pronombres	Residencia	Procedencia	Trabajo/estudio
Matías	16	Varón trans	Masculino	Unquillo, Córdoba	Unquillo, Córdoba	Estudia en una escuela secundaria pública.
Elodia	27	Mujer trans	Femenino	Córdoba Capital	Perú	Trabaja y estudia en la Universidad Nacional de Córdoba.
Niña X	31	Mujer trans	Femenino	Unquillo, Córdoba	Unquillo, Córdoba	Trabajadora sexual. Está terminando la escuela secundaria en un CENMA.
Noa	31	Varón trans	Masculino/No binario	Córdoba Capital	Salta	Licenciado en Psicología. Trabaja en la universidad y en consultorio clínico. Realiza investigación y se encuentra estudiando un doctorado.
Paz	43	Mujer trans	Femenino	Córdoba Capital	Gral. Mosconi, Salta	Trabaja en una carpintería y, eventualmente, como trabajadora sexual. Está terminando la escuela secundaria en un CENMA. Participa en una asociación civil.
Fede	31	Varón trans	Masculino	Córdoba Capital	Santiago del Estero	Trabaja como empleado en un banco, ingresó por Ley de Cupo Laboral Trans.
Vale	31	Mujer trans	Femenino	Córdoba Capital	San Juan	Trabaja como empleada en un banco, ingresó por Ley de Cupo Laboral Trans. Milita en una asociación y cursa una tecnicatura en una universidad privada.
Nacho	31	Varón trans	Masculino	La Calera, Córdoba	La Calera, Córdoba	Trabaja como empleado en Correo Argentino, ingresó por Ley de Cupo Laboral Trans.

Fuente: Elaboración propia. CENMA= Centro Educativo de Nivel Medio para personas Adultas Ley de Cupo Laboral Trans= Ley 27636 de Promoción del Acceso al Empleo Formal para personas Travestis, Transexuales y Transgénero.

A partir de las trayectorias, se identificaron tres grandes temas: 1) Proceso de transición; 2) Salud integral; 3) Impacto del contexto social, histórico y político en la salud.

### Proceso de transición

A partir de los relatos puede identificarse que las edades de inicio de los procesos de construcción corporal son mayoritariamente en la adolescencia, en ocasiones luego de alejarse de la familia de origen. En los inicios de los procesos de construcción corporal, varias personas entrevistadas, especialmente mujeres trans, comentan que comenzaron a tomar hormonas sin prescripción médica, controles ni acompañamiento profesional y/o a inyectarse silicona, con consecuencias negativas para la propia salud. Por ejemplo, Vale comenzó a los 13 años a consumir pastillas anticonceptivas que les quitaba a escondidas a sus hermanas y durante 10 años continuó utilizando hormonas sin prescripción médica. Niña X también refiere que desde chica comenzó a inyectarse hormonas que compraba en la farmacia por recomendación de otras mujeres trans: “*Las travestis grandes ellas te decían ‘vas te compras, te pones dos inyecciones a la semana’”* (Niña X, mujer trans, 31 años). 

Con relación a los inicios del proceso de construcción corporal, mientras en los relatos de las mujeres trans entrevistadas había una ausencia de referencias a profesionales de la salud, diferente es lo observado en los relatos de los varones trans, cuyos inicios fueron a partir de consultas con profesionales de la salud. Es importante mencionar el momento histórico en el que iniciaron sus procesos las mujeres trans entrevistadas -entre el año 2000 y 2010- cuando no existían espacios de salud visibilizados para la atención integral de personas trans. En cambio, los varones trans, a excepción de Fede, iniciaron luego de 2020, donde ya se contaba con estrategias sanitarias para la atención de salud de personas trans. Sin embargo, más allá de las diferencias en los inicios de los procesos entre varones y mujeres trans, en todos los relatos se evidencia la transmisión de experiencias y de información sobre la salud entre personas trans, así como la importancia de las redes para acceder a las hormonas, lo que destaca el aspecto relacional de la accesibilidad y la agencia de las personas trans para acceder a la atención en salud y a los procesos de construc­ción corporal. 

Asimismo, se observan diferencias generacionales. Por ejemplo, Matías, que inició su proceso en el año 2021 con 15 años, a casi diez años de la implementación de la Ley de Identidad de Género, contó con hormonas suministradas por el centro de atención primaria de salud de manera gratuita, y con posibilidad de seguimiento médico semanalmente; mientras que Fede, en 2011, si bien tuvo su primera consulta con una profesional médica, luego debió recurrir a redes informales para poder acceder a la medicación, y las utilizó sin seguimiento profesional. Esto también puede analizarse desde el enfoque biográfico, como resultado del impacto de las políticas públicas vigentes en cada uno de los momentos de inicios de procesos.

#### La importancia de las redes y del acceso a la información

Dentro de las trayectorias de vida de cada una de las personas entrevistadas hay momentos que pueden identificarse como puntos de bifurcación, conceptualizados desde el enfoque biográfico, que se vinculan con conflictos o situaciones de violencias, al interior de sus familias de origen, al visibilizar su identidad u orientación sexual en la adolescencia. En todas las experiencias de vida relatadas, a excepción de Matías que es la única de las personas que se encuentra atravesando la adolescencia al momento de la entrevista y que cuenta con el apoyo familiar y de pares, el resto tuvo que alejarse de sus familias de origen para poder expresar libremente su identidad y acceder a procesos de construcción corporal y/o a redes de apoyo. 

Estas desafiliaciones tempranas se constituyen en puntos de bifurcación en las trayectorias de vida, determinando un giro trascendental en sus vidas. En el relato de Paz, puede observarse que a los 17 años decide irse de su lugar de origen:

“...*me fui a Buenos Aires sin conocer y así ajjjaj, como el chavo del ocho con el bolsito y nos vemos... así fue*. [...] *Me fui la primera vez porque quería independizarme de mis cosas, conocer, no podía vivir con mi familia por cosas que yo quería hacer y mi familia no me dejaba, mi padre principalmente... y bueno decidí hacerlo lejos y me fui a Buenos Aires, y por esa razón me alejé de la familia y de Salta*”. (Paz, mujer trans, 43 años)

En el relato de Fede refiere:

“.*..el hecho de decidir irme a Buenos Aires tuvo vinculación con eso. Yo dije ‘yo rompo acá, rompo el nido, no lo entienden, en algún momento lo entenderán. Yo me voy y si en algún momento mi vieja toma conciencia nos volveremos a hablar y si no, bueno, yo estoy eligiendo por mí, es lo que yo quiero y listo’. Me planté con 18 años y me tomé el palo. Me fui de hecho sin despedirme de mi mamá, sin hablar...*”. (Fede, varón trans, 31 años)

Estos alejamientos implicaron distanciamientos geográficos, por lo que se movilizaron a ciudades en las que consideraban que sería posible acceder a proceso de construcción corporal y/o a contactos con organizaciones o personas trans, como así también a posibilidades laborales. En algunos casos, la movilización geográfica tuvo que ver con recurrir a la familia extensa que contenía y no violentaba ante la orientación sexual no heterosexual o la ya autoreconocida transexualidad.

A pesar de las hostilidades de la familia de origen, en todos los casos hubo alguien de la familia extensa (en general, abuelas o abuelos), que acompañó desde la comprensión y el cuidado. Vale relata el apoyo que recibió de su abuela:

“*...mi abuela, la mamá de mi papá que ya falleció, ella era una visionaria de lo que yo iba a ser. Es la que me enseñó a bordar, cocinar, me enseñó a pintarme, a maquillarme. Mi abuela me dejaba que yo me vistiera de nena en su casa y me dejaba jugar con sus cosas, porque ella sabía que yo era feliz, entonces mi abuela me dejaba hacer todo. Era un amor, siempre mi abuelita me ayudaba y me decía ‘juga con todo, acá tenés tu ropita’, me juntaba sus pinturas (se emociona), yo siempre digo que si estuviese viva yo creo que estaría re orgullosa, porque era una genia y siempre a mis papás hablándoles para que ellos pudieran ser más accesibles conmigo, como ‘escuchala, no le pegues, habla’*”*.* (Vale, mujer trans, 31 años)

En la mayoría de las trayectorias, quienes se habían alejado de sus familias de origen, luego de períodos prolongados de tiempo, fueron generando revinculaciones. En algunos relatos, la revinculación con la familia de origen aparece como un punto de bifurcación, a modo de reparación. Por ejemplo, Fede refiere en su relato:

“...*mi vieja lo fue entendiendo y después cuando volví a Santiago era ella la que me compraba cuando aprovechábamos, por el amigo farmacéutico, las hormonas.* [...] *cuando me hice la cirugía fue ella quien durmió en una reposera al lado de la cama, que me hizo las curaciones, que estuvo digamos en lo cotidiano, bancando todo. Y hoy tenemos la mejor. De hecho, hoy re apaña inclusive a personas trans que conoce allá, yo le hago el nexo o había momentos en los que le mandaba papeles o info y mi vieja andaba ahí haciendo de intermediaria como si nada y acá lo mismo con mis amigos la mejor, ni un drama*”. (Fede, varón trans, 31 años)

Ante la desafiliación, surgen nuevas redes que acompañaron procesos de transición, entre ellas se destacan otras personas trans, organizaciones sociales y espacios terapéuticos. En el caso de los varones trans, las amistades, parejas y exparejas son redes importantes mencionadas. Por ejemplo, Fede expresa que:

“*Pero por suerte siempre tuve amigos a la par, que estuvieron también desde el desconocimiento, de decirme ‘che ¿qué puedo hacer?’, y yo tener que dar un poco las indicaciones* [...] *Hoy estoy en pareja y siempre me dice ‘decime si vos necesitas, voy estoy’, se re preocupa de estas cuestiones y de las tensiones en el sistema*”. (Fede, varón trans, 31 años)

La vinculación con las organizaciones sociales que trabajan con temáticas trans y/o diversidades son relevantes como espacios de contención, escucha, circulación de información y solidaridad, tanto para los varones trans entrevistados como para las mujeres trans entrevistadas, facilitando la accesibilidad. Por ejemplo, para Nacho, otros varones trans pertenecientes a una organización social fueron claves para asesorarse y acceder a las consultas y a cuidados postoperatorios.

En los relatos resalta la importancia del acceso a información, ya sea respecto de la identidad de género trans, como de instituciones y profesionales considerados amigables para iniciar procesos de construcción corporal e incluso para cualquier otra necesidad de salud. Algunas personas entrevistadas refieren que la posibilidad de conocer vivencias de personas trans fue lo que les permitió poner en palabras y nombrar la autopercepción de la identidad, en los momentos fundantes de su transición, así como aprender un saber hacer transmitido oralmente. Por ejemplo, las mujeres trans destacan la información que recibieron de otras mujeres trans mayores que ellas, conformando lo que Vale menciona como “tejes”:

“*…tejes le llamamos a encontrarnos con nosotras mismas… a tenernos que tejear… no sé cómo decirlo, si te pones algo nunca te va a quedar así perfecto, entonces nosotras tenemos que agarrar, meterlo, hacerlo a nuestra forma, a nuestra manera para sentirnos cómodas*”. (Vale, mujer trans, 31 años)

Vale hace referencia a una peluquería atendida por una mujer trans a la que su abuela la llevaba cuando era chica:

“*En esa peluquería, el día viernes era el día de cotorreo, entonces caían todas las que vivían alrededor* [mujeres trans]*. Y entonces yo caía y estaba tan feliz porque eran todas tan divertidas que yo me mataba de la risa. Yo me iba tan chocha y feliz a mi casa. Y con ellas era con las que ibas aprendiendo… yo les decía ‘¿cómo hiciste para tener esa cola, esas lolas, el pelo?’”.* (Vale, mujer trans, 31 años)

Otras fuentes de información que se mencionan son el contacto o la participación en organizaciones sociales que brindaron espacios de contención e intercambio de saberes, principalmente en los inicios de los procesos, no solo a las personas protagonistas sino también a sus familias. Destaca también, especialmente entre varones trans, las redes sociales e Internet, un espacio virtual donde se genera un intercambio de opiniones y experiencias que les otorga sentido de comunidad. El uso de las redes sociales es una fuente de referencia para observar, a partir de la realización de videos/registros, los cambios corporales que genera el uso de hormonas. Matías lo expresa así: “*yo sigo a varios chicos trans de otros lados, de otros países, y he visto sus cambios y es bastante satisfactorio ver el cambio ese”* (Matías, varón trans, 16 años)*.*

También la creación de comunidades virtuales permite, como dice Fede:

“*Manejamos información sobre profesionales, o sea los pibes se van aconsejando sobre la experiencia con el médico que cada uno tiene, van contando cómo les fue, qué les pareció, en qué clínicas u hospitales están entregando hormonas, en donde están haciendo cirugías, con quienes tuvieron buenas experiencias, con quienes no. Toda esa información va circulando todo el tiempo y se va actualizando permanentemente* [en un grupo de Facebook]”. (Fede, varón trans, 31 años)

#### Relación con el sistema de salud ante el proceso de construcción corporal

De las personas entrevistadas, tres varones trans han atravesado mastectomías y dos mujeres trans se han realizado cirugías en el rostro y de implantes. La mayoría de las prácticas fueron en el subsector privado, a excepción de Nacho quien lo realizó en el subsector público. Con respecto a los tratamientos de hormonización, a excepción de Elodia, las personas participantes han realizado o se encuentran realizando tratamiento hormonal, la mayoría en el subsector público.

Los relatos vinculados al estigma, los malos tratos y la discriminación en el sistema de salud fueron frecuentes, aunque no fueron referidos como principal barrera. Sí se identifica como un obstáculo importante la escasez de profesionales y equipos especializados para tratamientos de hormonización y prácticas quirúrgicas. Noa relata:

“*Yo ya tenía fecha para hacerme la cirugía y después cuando voy con esta persona, la pase muy mal, te juro horrible porque no me quiso operar por gordo… Me dice ‘anda a un gimnasio todos los días y en tres meses te opero’. No, no, yo te juro que ese día fue tremendo y después de eso mand*é *una nota a* [nombre de la obra social] *solicitando que me atienda otra persona y me dijeron que no, que era la única persona*”. [Noa, varón trans, 31 años]

Los relatos resaltan la alta demanda y la sobresaturación de los espacios de salud, principalmente en el subsector público de la ciudad de Córdoba, donde hay largas listas de espera para acceder a prácticas quirúrgicas o a la atención de profesionales, especialmente, a aquellas personas profesionales identificadas como respetuosas. Si bien se reconoce que los nuevos espacios de salud considerados amigables son facilitadores, las personas participantes indican que son limitados con relación a la alta demanda actual de personas trans.

Otra barrera es la discontinuidad en la atención con las personas profesionales de la salud, con quienes se ha iniciado tratamiento, lo que se interpreta como una barrera en la accesibilidad ampliada, es decir, de permanencia en el proceso de atención de la salud. Ante la ausencia de la persona profesional con la que se inició el tratamiento, ya sea por licencias prolongadas o por retiro jubilatorio, se relatan experiencias en las que se abandona el tratamiento o se continúa sin seguimiento profesional. Paz, por ejemplo, luego de tres años de iniciado su proceso de hormonización, la médica que la atendía dejó de hacerlo, lo que implicó que Paz continúe su proceso sin acompañamiento profesional:

“*La doctora* [nombre de la doctora], *la que atendía acá, pero tuvo problemas ella y no atendió más así que estoy con las hormonas que me dio ella y sigo comprándolas por mi cuenta las que ella me recetaba*”. (Paz, mujer trans, 43 años)

Especialmente los varones trans señalan la poca o nula formación en salud trans de los equipos de salud, no solo con relación a prácticas quirúrgicas y tratamientos hormonales, sino también al acompañamiento personalizado e integral de la salud, constituyendo una barrera importante para su accesibilidad ampliada. Por ejemplo, se señala la ausencia de adecuación de los tratamientos a cada corporalidad y subjetividad. En las entrevistas, Nacho comenta cómo la falta de integralidad en la atención de salud para llevar adelante el proceso de hormonización implicó que desista de iniciarlo, lo que se evidencia como una barrera para el acceso ampliado, además destaca consecuencias en salud por su mastectomía:

*“…hace ya dos años que vengo entrenando el pecho, porque me qued*ó *hundido en los lados, para ver si generaba masa muscular, porque yo le pregunt*é *cuando me sac*ó *los drenajes que estaba como hundido, pero me dijo que se iba a acomodar… bueno, ella no es cirujana estética.* ¡*Es más! estaba aprendiendo, y bueno puede ser que pase y me tocó a mí... hay algunos chicos que les quedó una más alta que la otra, usaron hilos que no correspondía… después pasaba que en el postoperatorio te daban para dolor Actron que en realidad eso no te calma los dolores*”. (Nacho, varón trans, 31 años)

Señalan que las capacitaciones que se brindan al personal de salud del sector público sobre salud trans están focalizadas en el trato digno y respetuoso y no otros aspectos claves como mejorar la calidad de la atención en términos de conocimientos profesionales sobre tratamientos de construcción corporal, entre otros temas. Fede, sobre la base de su experiencia explica que:

“*…las aperturas están, pero siento que todos los médicos tienen la misma estructura. Porque* ¿*qui*é*n baja la capacitación?, o sea la capacitación que tienen en diversidad sexual es ‘respetemos pronombre, trans masculino es esto, trans feminidad es esto. Si viene una persona travesti es esto, si no tiene cambio registral lo ten*é*s que atender con el pronombre que se percibe’. O sea, esas son las capacitaciones…*”. (Fede, varón trans, 31 años)

Aparece también la incertidumbre ante la escasez de investigaciones y conocimiento sobre el impacto de los tratamientos hormonales de larga duración en sus cuerpos. Noa relata:

“*…tardé un tiempo en hormonarme, porque tenía a mi papá que tuvo cáncer… y en eso yo iba a comenzar a hormonizarme de una, pero tengo una prima que es médica, que está estudiando todo lo relacionado al cáncer y me advirtió que si tenía posibilidades de hacerme un estudio genético para ver si la influencia de la testo, en las consecuencias que puede haber en esto de tener antecedentes familiares y la predisposición a hacer eso, el uso de la testo. Por suerte me lo pudo costear, mi mamá, a ese estudio. Ahí me di con algo re interesante, digo interesante a la vez que me parece para pensar un montón y es que no hay investigaciones, porque como la mayoría de las personas trans no acceden a eso, de las consecuencias del uso de las hormonas en este caso la testo en los cuerpos*”. (Noa, varón trans, 31 años)

Esto también constituye una barrera en la accesibilidad ampliada, donde la desconfianza ante el desconocimiento de los impactos del uso prolongado de hormonas determina la interrupción de los tratamientos, muchas veces sin la intervención de profesionales. En el caso de Fede: “*...Y yo de cagón ya había dejado el tratamiento un poco por esto, la falta de estudios, no hay estadísticas... yo no sé qué va a pasar con mi cuerpo de acá a un tiempo*” (Fede, varón trans, 31 años).

Otro obstáculo identificado en el acceso a los procesos de transición es que, en general, no se ofrece una atención interdisciplinaria integral. Esto genera que las personas deban garantizarse la atención en salud mental o en ginecología, o medicina clínica u otras especialidades, en diversos espacios fragmentados, lo que aumenta su vulnerabilidad. A ello se suma, en algunos casos, la transición desde el subsector público al de obras sociales, a las cuales algunas personas tienen acceso -como Vale y tres de los cuatro varones trans entrevistados- a partir de su reciente inserción laboral formal, debido al cupo trans en algunos casos. Si bien esto aparece en los relatos como puntos de bifurcación en sus vidas, por las transformaciones positivas que implicó el acceso a un trabajo formal, y con ello a una obra social, esto les implica afrontar nuevos cambios y explorar nuevamente profesionales respetuosos, conocimiento que sí se disponían del sector público. Fede lo explica así:

“*…y ahora me está costando porque ahora tengo obra social y es como ‘chiquis yo siempre estuve en el público tírenme un dato de adónde voy en el privado’. Y es como que me estoy armando... a ver si empiezo a agarrar el listadito de todos los endocrinólogos que tengo por obra social e ir a golpear uno o uno, a ver con quienes tengo buenas experiencias*”. (Fede, varón trans, 31 años)

Para Nacho, la incertidumbre se deriva del desconocimiento sobre cómo es el acceso al tratamiento hormonal con la obra social que le corresponde por su inserción laboral formal:

“*Yo, por ejemplo, que ahora estoy trabajando y todavía no tengo la obra social, porque estoy decidiendo si lo tomo o no por el hecho de que no se si me va a cubrir el 100% del tratamiento. Yo hace tres años que estoy con hormonas y no lo quiero cortar. Yo me iba a afiliar a* [nombre de la obra social], *no lo hice porque me pidieron un informe con todos los análisis que tenía, que de todo bien, de la médica, la doctora* [nombre de la profesional] *me dijo que sí, que me va a hacer todos los análisis. Pero bueno, ellos me lo piden, pero yo no tengo la seguridad de que me lo vayan a dar. Entonces estoy tratando de ver cómo accedo a algún papel que realmente me diga que se comprometen a que me van a cumplir con la hormona*”. (Nacho, varón trans, 31 años)

Por otro lado, en los relatos se identifican estrategias, tanto individuales como colectivas, para acceder al sistema de salud. Destacan las experiencias colectivas de organización, principalmente compartir información, uso de las redes sociales, redistribución colectiva de hormonas entre varones trans y vinculación con organizaciones sociales. Por otro lado, algunas personas se sienten como “*conejo de indias*” ya que a través de la propia experiencia no solo identifican personas profesionales que son respetuosas, sino que contribuyen en la formación o marcan precedentes para la atención de otras personas trans. Fede lo expresa así:

“*Es un camino que va abriendo, de una u otra forma, o sea alguien lo inició, alguien fue el primer paciente de esa persona. No les enseñan en la universidad… Él* [médico cirujano que le realizó la mastectomía] *al principio no entendía nada… me empezó a pedir si tenía guías, folletos, cosas como para que él pudiera ir viendo, aprendiendo, entendiendo*”. (Fede, varón trans, 31 años)

Similar a Fede, Nacho menciona:

“*...creo que en la medicina siempre hay una instancia en donde es prueba y error y creo que así se llegan a los medicamentos, a curar enfermedades, hacer tratamientos. Y creo que estuvo en m*í *decir ‘bueno yo me mando’, ser conejo de indias en este momento. Pero es lo que toca, no me siento inconforme porque siento que, a raíz de eso, las siguientes cirugías fueron mejorando un poquito más, pero bueno así se empieza el camino en todo… o sea no me siento inconforme de haber dado ese paso*”. (Nacho, varón trans, 31 años)

Estas experiencias pueden interpretarse como estrategias que se implementan para aportar a disminuir las barreras en la accesibilidad ampliada, y ejemplifica el carácter relacional en la construcción de la accesibilidad. Esta predisposición a brindar información y conocimientos a profesionales, a partir de las propias experiencias, simboliza a su vez la relevancia del sentido de lo colectivo.

Los consultorios llamados inclusivos o amigables fueron destacados como facilitadores de acceso a tratamientos integrales, especialmente en las localidades cercanas a la ciudad, ya que ofrecen un trato más humano y horarios flexibles. Destacan también el rol de las personas promotoras en salud trans que integran dichos equipos y son quienes articulan con las organizaciones para garantizar derechos. Niña X estuvo desarrollando ese rol a partir de la pandemia y Matías menciona el efectivo acercamiento de la población trans al consultorio inclusivo en una localidad de Córdoba:

“*...los casos de chicos trans que fueron asistiendo ahí en el dispensario ha cambiado mucho entre la pandemia. Hay más casos de chicos trans acá en Unquillo que me han comentado, y de chicas trans también, que se acercan y se sienten más cómodos ahí en el dispensario*”. (Niña X, mujer trans, 31 años)

En este sentido, a partir de los relatos también puede observarse la accesibilidad como un proceso dinámico y relacional, que no solo depende de las decisiones de las instituciones y políticas de salud sino también se genera por las personas usuarias del sistema, tanto de forma individual como colectiva.

### Salud integral

Indagar sobre el concepto de salud permitió una aproximación a conocer cómo y qué priorizan y es relevante para el cuidado de su propia salud. Cabe destacar el énfasis en la salud mental y la necesidad de contar con acompañamiento psicológico, como un aspecto fundamental en la salud. Como expresa Niña X:

“*…la salud es todo, sin salud no vivimos. Es el motor del cuerpo… Tener una buena alimentación… si te hormoniz*á*s que te acompañen psicológicamente... porque la verdad es que transitar por un género… es algo que necesitás un apoyo de alguien. Y bueno, eso creo que en la salud sería primordial.* ¡*Acompañamiento a las chicas... y salud! jajaj* ¡*mucha salud!*”. (Niña X, mujer trans, 31 años)

Esta mención a la salud mental es una manifestación de la necesidad de dar relevancia a los espacios psicoterapéuticos como parte de los procesos de transición, no como un requisito u obligación, sino como un derecho para quienes lo deseen. Vale indica que:

“*La salud para m*í *es lo primordial para el ser humano, es lo más importante, sin salud no podemos hacer nada. Y nos dimos cuenta hace muy poco tiempo que es muy esencial en la vida de los seres humanos; y para nosotras las mujeres trans, sobre todo, es doblemente primordial. Porque si no contamos con una buena salud, podemos tener muchos problemas a causa de todas esas cosas de las adicciones, a causa de inyectarse aceite, a causa de la medicación, a causa de no poder ir al sistema de salud porque capaz est*á*s en un estado, o cuando tu familia te expulsa de tu casa y ejerc*é*s el trabajo sexual y ya capaz que te encontrás con este virus y no la peleas por más que ahora tomas una pastilla, sentís que ya nadie te va a querer y si no estas acompañada con psicólogo…*”. (Vale, mujer trans, 31 años)

Otro de los aspectos resaltados en las definiciones sobre la propia salud es la integralidad, en dos sentidos, el primero vinculado a la atención de los equipos de salud en torno al proceso de construcción corporal, y el segundo en vivenciar la propia salud como un bienestar que implica, además, el autocuidado y la toma de decisiones autónomas para sentirse bien. Por ejemplo, para Paz implica poder disfrutar de aspectos de la vida cotidiana y contar con un trabajo que le permite hacer lo que le gusta. Para Elodia la salud es un equilibrio, el balance entre lo externo y lo interno, así como también no ser discriminada en su contexto. Para Matías, quien se encuentra formando parte de un grupo de adolescentes convocado por el centro de atención primaria de salud de su barrio, y coordinado por agentes de salud y profesionales de salud, se puede ver cómo se trabaja el concepto de salud desde una mirada integral, que lo manifiesta con ejemplos concretos de su propia vida, a la hora de definir la salud:

“*Y ocuparme de mí mismo también fuera de lo que sería ir al médico todo eso… cuidarme.* [...] *Darme un respiro si siento que lo necesito… si estoy estudiando y estoy muy estresado dejar un rato y después seguir… No obligarme a hacer ciertas cosas… eh… hacer más lo que me gusta más que lo que quieran los demás*”. (Matías, varón trans, 16 años)

#### Barreras y estrategias para el acceso a la atención de la salud integral

Las históricas experiencias de discriminación y estigma en el sistema de salud vividas por personas trans operan como obstáculo para recurrir al sistema de salud ante cualquier problemática de salud. 

“*…yo al hospital no iba, a ningún hospital fui jamás… porque no me gustaba, porque siempre el prejuicio y una vez quise ir y fui como muy discriminada”.* (Niña X, mujer trans, 31 años)

“...*la verdad que evito mucho ir a los médicos… llegue a ese punto que los controles que hago los hago obligatoriamente porque no queda otra y acudo al sistema de salud cuando hay una dolencia*”. (Fede, varón trans, 31 años)

“*...no ten*é*s derecho a enfermarte porque... ¿a dónde vas?... O no te atienden o te ven la cara y te quieren cobrar… te tratan como masculino, o sea, la gente sabe lo que a una le molesta y te mete el dedo en la llaga y hace eso para molestarte*”. (Paz, mujer trans, 43 años)

Por otro lado, destacan un sesgo en la atención vinculado a asignar el uso de hormonas como causa o como coadyuvante de cualquier malestar de salud. Fede lo relata así:

“*Vas a un médico porque te duele la panza y es la hormona, vas a un médico porque te doblaste el brazo y es ‘se te descalcificó el hueso por la hormona’*”. (Fede, varón trans, 31 años)

Como estrategia para recibir una mejor atención, algunos varones trans relatan no mencionar su identidad trans. Fede comenta que:

“*Por eso pasa que muchas personas trans hoy deciden no decir que son trans en una consulta, si no es necesario, y ahí mágicamente no te duele el hombro por ser trans, sino porque te golpeaste. Yo eso lo re probé, a mí me encanta hacer esas pruebas*”. (Fede, varón trans, 31 años)

Otro de los obstáculos recurrentes relatados por varones trans ocurre en el acceso a las consultas ginecológicas, donde aparecen experiencias de discriminación y estigma, no solo por no reconocer su identidad autopercibida, sino por la discriminación sufrida al no atender demandas de salud que presentan. También destacan obstáculos en el acceso a turnos. Fede explica que:

“*He tenido compañeros que no pudieron acceder a turnos de ginecología por la obra social, porque las planillas están armadas de determinadas formas que solo el género femenino tiene disponibilidad de sacar un turno, no así las masculinidades. Entonces si vos te afilias a una obra social como Fede y querés sacar un turno con ginecología por una página web, no te da la opción, y si lo vas a sacar personalmente o presencialmente, los formularios también están hechos en femenino”.* (Fede, varón trans, 31 años)

En ese sentido, se mencionan estrategias de acceso a consultas ginecológicas como, por ejemplo, acceder a centros de salud y no a hospitales o ingresar a alguna consulta como acompañante de otra persona, como relata Matías: “*fui a acompañarla a mi hermana y entré con ella y para sacarme la duda le pregunté a la ginecóloga*” (Matías, varón trans, 16 años).

Al igual que en el proceso de transición, la circulación de información sobre espacios y profesionales considerados amigables, ya sea a través de pares o por referencias de profesionales de la salud con quienes ya vienen realizando tratamiento, es otra estrategia colectiva de acceso. En el caso de las consultas ginecológicas, esta red de referencias se amplía, incluyendo a mujeres cis. Acudir con el acompañamiento de alguna persona de confianza aporta seguridad y resguardo, especialmente, en situaciones consideradas de mayor gravedad o posibilidad de discriminación. Sin embargo, en general las personas refieren que acuden solas a las consultas de salud.

Otra estrategia de acceso destacada, especialmente entre mujeres trans, son las organizaciones sociales ya que generan articulaciones con instituciones y equipos de salud para poder garantizar un acercamiento de las personas trans a diferentes especialidades médicas o incluso algunas tienen consultorios propios. En la experiencia relatada por Paz se encuentra la Asociación de Travestis, Transexuales y Transgéneros de Argentina (ATTTA), en Córdoba Capital, donde en la misma sede cuentan con consultorio médico y prácticas diversas que articulan, a su vez, con hospitales públicos para facilitar el acceso a turnos considerados protegidos. En este sentido, estas estrategias facilitan el acceso, tanto inicial como ampliado, y las organizaciones y colectivos operan como intermediarios que cuidan y garantizan atención en salud. También se menciona el nexo con el equipo de salud municipal y con el equipo del área de género municipal de una localidad cercana a la capital, desde donde se facilita el acceso a turnos, prácticas y a medicación, ya sea en la misma localidad o en la ciudad de Córdoba, en caso de no contar con la especialidad o insumos requeridos. Niña X lo expresa así:

“*…siempre, para cualquier problema, ellas ahí no más ‘venite, llegate’. La mínima cosa que me pasa, les digo a ellas, medicamentos y algo me dicen ‘venite, te vemos’... Por eso, desde ahí empecé a ir a los centros de salud de ac*á*... gracias a las compañeras… que nos dan un turno... y dec*í*s ‘¡Guauuu!* ¡¡*que alivio!!*’”. (Niña X, mujer trans, 31 años)

Por último, como estrategia de acceso, uno de los varones trans entrevistado menciona la pertenencia a un grupo en el centro de atención primaria de salud de su barrio que, si bien no se conformó específicamente para personas trans, se presenta como una estrategia comunitaria de salud, propuesta desde la propia institución, con la apertura para trabajar temáticas de sexualidad y adolescencias. Este grupo se constituye en un espacio que contiene y que además es referencia para el acceso a prácticas o consultas mediante el vínculo con las personas que coordinan el grupo, agentes sanitarias, psicólogas, médicas, etc. En este sentido, se considera esta experiencia como red que acompaña en torno a la salud integral, entendiendo que estos espacios de referencia alojan y contienen, a la vez que fortalecen vínculos entre pares adolescentes a través de la construcción de autonomías y de identidades desde las propias potencialidades. A su vez, la asiduidad al centro de salud para las reuniones semanales actúa como facilitador para el acceso.

### Impacto del contexto social, histórico y político en la salud

#### Cambios a partir de la Ley de Identidad de Género

Los relatos destacan cambios positivos a partir de la sanción de la Ley 26743 de Identidad de Género ya que posibilitó el acceso a derechos para las personas trans, principalmente en los últimos años. Según la mirada de Vale sobre la ley, hubo mejoras en el ámbito de la salud, donde reconoce un avance en cuanto a la información y conocimientos disponibles que favorecen vivenciar la identidad y acceder a procesos de transición:

“*…después de la Ley de Identidad de G*énero hubo otra perspectiva, los médicos tomaron otras referencias y buscaron más *agiornarse respecto a lo que ellos ya venían viendo por décadas. M*édicos más grandes, ellos dicen, existieron siempre las chicas trans, pero no había esa información que hay ahora, que se pueden hacer un montonazo de cirugías y cuestiones con respecto a la medicina y poder lograr con una chiquita a temprana edad facilitarle si quiere cambiar su género”. (Vale, mujer trans, 31 años)

En los relatos se destaca que, a partir de la ley, hay equipos que atienden de manera integral la salud trans, principalmente, en el subsector público, lo que mejora el acceso, tanto inicial como ampliado. Fede resalta la importancia de la autopercepción de la identidad para el acceso:

“Antes de la Ley de Identidad, como no había ley, la medicina lo que hacía era decirte ‘bueno está bien, nosotros te hacemos hacer el tratamiento hormonal pero vos necesitas sí o sí tener un apto psiquiátrico que contemple esto porque es como una enfermedad’, era todo el drama de la disforia”. (Fede, varón trans, 31 años)

El acceso al cambio registral es destacado en todos los relatos. En el caso de Elodia menciona que: “...*la Ley lo que permite es… que seamos considerados seres humanos*” (Elodia, mujer trans, 27 años)*.* Menciona que el acceso al cambio registral permite también la posibilidad de realizar denuncias ante violencias. Su mirada positiva sobre los cambios generados por la ley es relativizada en tanto considera clave que la ley se acompañe de un cambio cultural. Por ejemplo, menciona que el respeto aún depende de cuánto las personas trans se acerquen a un ideal hegemónico:

“*...no es lo mismo si sos una piba trans rubia, de ojos claros, con una corporalidad hegemónica, que si no lo sos, que si sos pobre, que si sos... no sé, de otra contextura física… Yo te puedo decir que ‘¡ay s*í*!* ¡*me tratan re bien!* ¡*veo un re cambio en la sociedad!’* Sí, porque estoy siguiendo y jugando este juego, porque entendí que jugando ese juego me iban a violentar menos”. (Elodia, mujer trans, 27 años)

Otros relatos, tanto de varones como de mujeres trans, consideran que faltan muchos cambios aún por concretar, en acceso a la vivienda y al trabajo. Fede hace una crítica sobre aspectos que no fueron contemplados en el momento de la sanción y que, luego de más de una década, no han sido resueltos:

“*…en el cambio registral, las personas que tienen hijos tienen el drama con la partida de nacimiento… quedó el hueco con el tema de los padrones electorales, con los CUIL* [Código Único de Identificación Laboral], *con el sistema de salud en general… las obras sociales se agarran de esto, del Plan M*édico *Obligatorio que cubre que no cubre… ten*é*s que declarar que sos una persona trans como si fuera una patología*”. (Fede, varón trans, 31 años)

Paz añade: “Si gracias a esa Ley, la identidad del documento, eso veo que sí, pero en otras cosas no, porque no tenemos vivienda, acceso fácilmente a la salud…”

#### Pandemia por covid-19 e impacto en la salud

La vivencia de la pandemia se observa con similitudes y diferencias para las personas entrevistadas que en ese momento residían en Córdoba Capital y quienes residían en otras localidades. En común, se enfatiza la consolidación de las redes entre organizaciones y partidos políticos, que generaban articulaciones y estrategias comunitarias para garantizar recursos e insumos básicos a las personas trans. Nacho recuerda que:

“*...en pandemia, la mayoría de las identidades trans es gente que no tenía un laburo formal o que tenía emprendimiento y estaban todos en ‘una’, nada nuevo, y fue como ponerse a armar bolsones, repartir, buscar alcohol, hicimos ropero comunitario... en ese periodo yo creo que paso muchas cosas*”. (Nacho, varón trans, 31 años)

Elodia destaca cómo se reforzaron redes previas y surgieron nuevos espacios para la población trans:

“*Sí, durante la pandemia me sorprendió la comunicación como la hemos usado y la red virtual que tejimos para poder llevar alimentos y todas esas cosas, eso sí me sorprendió. Esa red funcionó y bastante bien* [...] *Cómo se llegó a la mayoría de los barrios más aislados... muchos barrios… Y de esas estrategias en algunos espacios políticos han seguido… Esas estructuras se han quedado en ciertos movimientos políticos que se han reforzado y que incluso en sus espacios de encuentro de sus organizaciones han logrado construir comedores, han logrado construir incluso espacios de salud de testeos de VIH. ATTTA* [Asociación de Travestis, Transexuales y Transgéneros de Argentina] *incorporó el Centro de Contención Trans, creo que se llama, donde también hay endocrinología, dentistas, oculistas... En ese espacio, que es un espacio específico de la organización, se han abierto merenderos y esas cosas que han surgido por estas estrategias en la pandemia*”. (Elodia, mujer trans, 27 años)

Para las personas trans, que en el momento de la pandemia residían en Unquillo, el surgimiento de una organización social disidente que implicó mayor visibilización del colectivo LGBTTTIQ+ en la localidad, la conformación del consultorio inclusivo en un centro de atención primaria de salud dependiente del municipio, y la formación de promotoras trans de salud municipales fueron hitos claves. Todas estas instancias facilitaron el acceso a tratamientos hormonales mediante su distribución a nivel local. Previo al aislamiento social y preventivo obligatorio, las personas trans de Unquillo retiraban sus hormonas en un hospital de la capital. En este sentido, Matías destaca que:

“*…los casos de chicos trans que fueron asistiendo ahí en el dispensario ha cambiado mucho entre la pandemia. Hay más casos de chicos trans acá en Unquillo que me han comentado, y de chicas trans también, que se acercan y se sienten más cómodos ahí en el dispensario*”. (Matías, varón trans, 16 años)

La pandemia generó impactos en la vida de las personas trans entrevistadas. Si bien se evidencia la profundización de las vulneraciones de este colectivo, principalmente por problemas de salud mental vinculados a las incertidumbres y angustias por la interrupción de sus tratamientos hormonales^29^, también implicó el surgimiento de estrategias colectivas. Muchas de las personas trans comenzaron a participar de instancias colectivas de manera activa, como estrategias para el acceso a recursos, entre ellos el acceso, tanto inicial como ampliado, a la salud. En algunos de los relatos se expresa que comenzaron a recibir atenciones de salud que hasta ese momento no accedían. Niña X refiere sobre su formación como promotora de salud:

“*...empezamos en pandemia con las chicas. Hicimos la formación, estudiamos.* [...] *muchas chicas no iban a los hospitales, no iban a los centros de salud. Temas de enfermedades, cosas... las chicas se terminaban yendo a Córdoba a hacerse tratar, por ahí era un viaje ir, siendo que teníamos todo el recurso acá en Unquillo*”. (Niña X, mujer trans, 31 años)

## DISCUSIÓN

El objetivo del presente trabajo fue conocer la accesibilidad a los servicios de salud de las personas transgénero que residen en la provincia de Córdoba, a partir de sus trayectorias de vida, indagando tanto las barreras como las estrategias de acceso. Los resultados destacan que en los inicios de los procesos de transición hay soledad y ausencia de contención, tanto de redes familiares como de profesionales de la salud, especialmente en el contexto previo a la sanción de la Ley 26743 de Identidad de Género. Esta exclusión coincide con lo identificado en otros estudios ya que impacta significativamente en las trayectorias vitales de las personas trans^6^^,^^30^^,^^31^. Por ejemplo, el rechazo de familiares está asociado a una mayor posibilidad de suicidio y depresión^32^. Ante esta situación, las personas generan nuevas redes de referencia, que incluyen principalmente otras personas trans y organizaciones sociales. Encontrarse con personas que viven situaciones similares proporciona referencia, permite la socialización y el intercambio de experiencias y recursos^19^.

La transmisión de experiencias entre pares en torno a los cambios y a la construcción corporal en los procesos de transición es clave. Otros trabajos también identifican estas prácticas sobre todo en los inicios de los tratamientos^8^^,^^9^^,^^10^^,^^17^^,^^33^^,^^34^^,^^35^. Los motivos que llevan a sostener estos procesos de manera autoadministrada y sin la intervención de profesionales tienen que ver con las discriminaciones y malos tratos recibidos en el sistema de salud, que sigue siendo uno de los factores principales de obstáculos en la accesibilidad, al igual que fue identificado en nuestro estudio y que fue documentado por organizaciones travestis y trans. En los relatos de las mujeres trans aparecen las autocríticas sobre la automedicación y las prácticas sin acompañamiento profesional realizadas en el pasado, entre los años 2000 y 2010, previo a la sanción de la Ley de Identidad de Género, especialmente por el impacto en la salud. 

Al igual que en nuestros resultados, Zalazar *et al*. observan mayor disfrute de derechos en las nuevas generaciones, junto con una mayor aceptación social y una mayor inclusión en el sistema sanitario, en gran parte gracias a la legislación^33^. Como es reconocido, la Ley de Identidad de Género no solo otorgó un marco de legalidad a prácticas y saberes expertos de la población trans sino que contribuyó al reconocimiento de derechos^7^^,^^36^. Nuestros resultados destacan no solo el impacto de la Ley de Identidad de Género, al igual que otros estudios realizados en Argentina^6^^,^^7^^,^^33^^,^^37^^,^^38^, sino también de la Ley 27636 de Cupo Laboral Travesti-Trans en el acceso a la salud, especialmente mediante el acceso a obras sociales. 

A pesar de los avances, nuestro estudio muestra que aún persiste una brecha entre la letra de la ley y las prácticas y discursos discriminatorios aún presentes en instituciones de salud^39^^,^^40^. En ese sentido, se observa que persiste una reticencia a acudir a consultas de salud, que revelan la permanencia de barreras simbólicas y culturales identificadas en otros estudios^5^^,^^6^^,^^12^^,^^33^^,^^41^, especialmente en el sector de obras sociales, ya que la información sobre profesionales amigables identificados está referida principalmente al sector público^6^^,^^31^^,^^42^. A esta barrera, se suman los turnos limitados, las largas listas de espera, la escasez de profesionales, la discontinuidad de las atenciones con profesionales con quienes se había iniciado tratamiento, barreras identificadas como contextuales^33^.

En la atención de la salud trans se ha identificado la coexistencia de dos modelos: los grupos interdisciplinarios coordinados de modo centralizado, los llamados consultorios inclusivos, y personas profesionales sensibilizadas que ejercen de manera aislada^36^^,^^43^. Las críticas al primer modelo señalan que la atención centralizada en espacios exclusivos para personas trans limita las acciones de visibilidad y exigibilidad de derechos en el resto del sistema de salud^44^. Sin embargo, en nuestros resultados este aspecto no ha sido señalado. Por el contrario, nuestro estudio destaca la importancia del consultorio inclusivo de una localidad cercana a la localidad cordobesa, que facilitó el acceso a tratamientos integrales, y permitió el acercamiento de personas trans a la salud, especialmente después de la pandemia. 

Si bien la segmentación y la fragmentación del sistema sanitario plantean desafíos para la integralidad y el cuidado de la salud de toda la población^45^, en las personas trans, circular por diferentes espacios de salud para garantizar el cuidado de la salud integral podría implicar mayores vulnerabilidades ante las discriminaciones y estigmas. En cuanto a las personas profesionales sensibilizadas, nuestros resultados revelan la importancia de la información entre pares. En general, las personas trans suelen llegar a consultas en el sistema de salud, con referencias previas, lo que facilita la accesibilidad. Esto se observa también en Zalazar *et al*.^33^, con las experiencias relatadas por mujeres trans. 

Otra estrategia identificada es acudir a las consultas con el acompañamiento de personas de confianza, lo que también se observa en otro estudio lrealizado en la provincia de Córdoba^42^. En las trayectorias de los colectivos y personas trans ocurre una acumulación de información. Se construyen conocimientos en torno a la salud a partir de las propias experiencias de salud y con el sistema de salud, y las personas son expertas en su propia salud^29^, lo que mejora la accesibilidad, la propia comprensión de la salud y la toma de decisiones^38^*.* A partir de este bagaje de conocimientos, el encuentro con nuevas personas profesionales es visto como una oportunidad para sensibilizar e incidir en su formación, según refieren algunas de las personas trans entrevistadas. En este sentido, puede entenderse la accesibilidad como un proceso dinámico, que no solo depende de las decisiones de las instituciones y de las políticas de salud, sino que también evidencia cómo la accesibilidad es generada también por las personas usuarias del sistema^23^. 

Las organizaciones sociales son también claves en la accesibilidad, tanto como espacios de circulación de la información sobre la salud como en la articulación entre sectores^42^, especialmente fortalecidas en la pandemia^29^. Al igual que en otros trabajos, la importancia de las redes sociales es destacada no solo por la transmisión de información, sino como espacios de sociabilidad para construir y fortalecer sus redes sociales de apoyo^29^^,^^46^.

Las críticas al sistema médico producidas desde miradas trans cuestionan el carácter cisexista de las prácticas de atención en salud y los procesos de normalización del género y la sexualidad producidos desde las instituciones sanitarias^29^^,^^36^. En ese sentido, nuestros resultados también cuestionan la formación profesional^42^^,^^44^, especialmente por parte de los varones trans. Anahí Farji Neer indica que continúa siendo muy escasa la incorporación de temas vinculados a la salud trans en la currícula de la formación universitaria, es por ello que la formación de las personas profesionales suele ser de manera autodidacta y voluntaria^5^. Otro de los aspectos cuestionados es la escasez de investigaciones sobre salud trans, especialmente sobre las consecuencias de los tratamientos hormonales^29^^,^^47^.

Por último, destacan las organizaciones sociales como facilitadores a través de las articulaciones con instituciones de salud y el Estado^42^^,^^48^, especialmente en el contexto de pandemia^29^^,^^49^^,^^50^. Otra estrategia identificada fue la formación de promotoras de salud trans y su incorporación al equipo del consultorio inclusivo, ya que permitió un acercamiento entre la comunidad, a través de las organizaciones de la sociedad civil o referentes, y el sistema de salud^33^^,^^37^*.*

## CONCLUSIONES

En primer lugar, el reconocimiento de la identidad autopercibida implicó situaciones de tensión, conflicto y, en muchos casos, violencias en los núcleos familiares cercanos. Cabe resaltar que, en la actualidad, se podría esperar que las adolescencias trans cuenten con otras posibilidades de aceptación y transición, por los avances legales, sociales y culturales. 

En segundo lugar, si bien se realizó una distinción de las experiencias con el sistema de salud vinculadas al proceso de construcción corporal y a la salud integral, esto es indivisible. La identidad de género atraviesa todas las aristas del sistema, ante cualquier necesidad vinculada a la salud; particularmente, en torno a las discriminaciones que continúan operando y limitando el acceso sobre todo para las mujeres trans, a la falta de conocimientos sobre las experiencias corporales y subjetivas, como así también a los impactos en la salud de las transformaciones corporales. La mayor demanda, entonces, tiene que ver con la falta de conocimientos de los equipos y la integralidad para el abordaje de la salud. Esto plantea desafíos tanto para la formación académica de profesionales de la salud, como para la exigibilidad de actualizaciones técnicas y científicas para equipos de las instituciones de todo el sistema de salud que deberían ser consideradas.

Como tercer punto destacable, se puede concluir a partir de los resultados obtenidos que la accesibilidad al sistema de salud fue mejorando, principalmente, a partir de los últimos años. Ejemplo de ello es la actual multiplicación de los denominados consultorios inclusivos en la provincia de Córdoba, que ponen a disposición equipos interdisciplinarios especializados, donde muchos de ellos cuentan con promotoras y promotores trans que facilitan la llegada a los espacios, como así también programas estatales específicos para el acceso a la salud trans y al trato digno. Estos son algunos de los aspectos en términos de avances que se pueden valorar. Sin embargo, persisten las barreras administrativas, culturales y simbólicas que deben ser atendidas.

En cuarto lugar, si bien existen diferencias entre las experiencias de los varones trans y las mujeres trans, como así también en términos generacionales, en todas las trayectorias las organizaciones sociales y las personas pares fueron y son claves para las transiciones. La contención, el asesoramiento, la posibilidad de brindar recursos e incluso para generar articulaciones con instituciones de salud, son fundamentales para garantizar el acceso, y así ha sido históricamente. La experiencia colectiva genera, además, las aperturas a nuevos vínculos entre pares y también con personas trans más grandes en términos de edad, como un entramado en el que se va transmitiendo la historia de luchas y conquistas del colectivo, como así también las deudas pendientes para garantizar la igualdad de derechos.

El intercambio de información a través de las redes puede ir cambiando de nombres y de modalidades, pero subsisten a lo largo de todas las trayectorias relatadas. El acceso a Internet, claramente, ha propiciado una mayor expansión, llegando principalmente a personas trans que se encuentran más alejadas de las grandes ciudades. El acceso a las redes sociales permite conocer experiencias con las cuales se identifican y reconocen.

En quinto lugar, la construcción de saberes y conocimientos, colectivos e individuales, a partir de las experiencias acumuladas actúa como generadora de accesibilidad, construida desde las propias personas que requieren prácticas de salud. Las vivencias de las personas trans aportan conocimientos a las y los profesionales sobre el proceso de transición, no solo en aspectos técnicos, sino también en cuanto a las experiencias y sentires vinculados a su salud entendida integralmente. 

Nuestro estudio muestra que la accesibilidad a la salud para las personas trans en Córdoba es un proceso dinámico, en el que interactúan el Estado provincial y municipal, las organizaciones sociales y las personas usuarias del sistema, en un intercambio permanente de información y conocimientos. En ese proceso, se pueden observar avances, a la vez que se evidencian la persistencia de barreras históricas que son necesarias y urgentes de superar. La participación organizada de la población trans en la incidencia y generación de la accesibilidad a la salud es imprescindible, al igual que la generación de políticas públicas sanitarias, y la defensa y actualización de las leyes que otorgan garantía de derechos a las personas trans.
